# Comparative Analysis of Postoperative Complications of Sentinel Node Identification Using the SentiMag^®^ Method and the Use of a Radiotracer in Patients with Breast Cancer

**DOI:** 10.3390/curroncol29050235

**Published:** 2022-04-19

**Authors:** Andrzej Lorek, Katarzyna Steinhof-Radwańska, Wojciech Zarębski, Joanna Lorek, Zoran Stojčev, Jacek Zych, Aleksandra Syrkiewicz, Paweł Niemiec, Karol Szyluk

**Affiliations:** 1 Department of Oncological Surgery, Kornel Gibiński Independent Public Central Clinical Hospital, Medical University of Silesia in Katowice, 40-514 Katowice, Poland; wzarebski@sum.edu.pl; 2 Department of Radiology and Nuclear Medicine, Kornel Gibiński Independent Public Central Clinical Hospital, Medical University of Silesia in Katowice, 40-752 Katowice, Poland; mammografia@uck.katowice.pl; 3 Department of Surgery, Ludwig Rydygier Hospital sp. z.o.o., 31-826 Krakow, Poland; asja.lorek@gmail.com; 4 Teaching Department of Oncology and Breast Diseases, Central Teaching Hospital of the Ministry of Internal Affairs and Administration, Wołoska 137, 02-507 Warsaw, Poland; stojcevz@gmail.com; 5 Medical Faculty, Medical University of Silesia in Katowice, 40-514 Katowice, Poland; jack.zych@gmail.com (J.Z.); alorys2112@o2.pl (A.S.); 6 Department of Biochemistry and Medical Genetics, School of Health Sciences, Medical University of Silesia in Katowice, 40-752 Katowice, Poland; pniemiec@sum.edu.pl; 7 Department of Physiotherapy, Faculty of Health Sciences in Katowice, Medical University of Silesia in Katowice, 40-752 KatowiSce, Poland; karol.szyluk@sum.edu.pl; 8 Department of Orthopaedic and Trauma Surgery, District Hospital of Orthopaedics and Trauma Surgery, 41-940 Piekary Śląskie, Poland

**Keywords:** sentinel lymph node biopsy, superparamagnetic particles of iron oxide, SentiMag^®^, breast cancer, complications

## Abstract

(1) Background: The purpose of the study was a retrospective, comparative assessment of complications of the surgical sentinel node biopsy (SNB) procedure in breast cancer using the radiotracer method and the SentiMag^®^ method on groups of patients after 3.5 years of use. (2) Methods: The material was a group of 345 patients with primary surgical breast cancer who underwent the SNB procedure with the use of a radiotracer in combination with wide local excision (WLE), simple amputation (SA) with SNB and an independent SNB procedure in the period from May 2018 to January 2021 in the Department of Oncological Surgery. Of the patients who were monitored in the Hospital Outpatient Clinic, 300 were enrolled. The analyzed group was compared in terms of the occurrence of the same complications with the group of 303 patients also operated on in our center in the period from January 2014 to September 2017, in which SN identification was performed using the SentiMag^®^ method. (3) Results: The most common complications found were sensation disorders in the arm, which occurred in 16 (14.1%) patients using the radiotracer method, SentiMag^®^-11 (9.9%). By comparing the complication rate between the methods with the radiotracer (n = 300) and SentiMag^®^ (n = 303), no significant differences were found. (4) Conclusions: Sentinel node (SN) identification using the radiotracer method and the SentiMag^®^ method are comparable diagnostic methods in breast cancer, with a low risk of complications.

## 1. Introduction

Surgical sentinel node biopsy (SNB) is currently the standard procedure for assessing regional lymphatic drainage in patients with diagnosed breast cancer without metastases. The procedure allows for a reduction in the number of lymphadenectomies performed, reducing the risk of complications. [1,2,3,4] Its use in patients with the clinical feature of N0 allows for the reliable assessment of lymph nodes, with a low risk of recurrence in the event of a negative result [5,6]. The most frequently used reference method is the method with a radiotracer, most often Technetium-99, performed alone or as a dual method in combination with a stain. Postoperative complications related to their use may include allergic reactions, permanent tattooing, limitation of limb mobility and lymphoedema [7,8]. The use of an isotope requires access to a nuclear medicine facility and is associated with a fast half-life, and the use of an isotope is not indifferent to both the patient and the team performing the study. An alternative method is the SentiMag^®^ ferromagnetic method, which uses the Sienna +^®^ colloid, which is a suspension of superparamagnetic iron oxide (SPIO) nanoparticles absorbed by the vessels of lymph nodes. Clinical studies performed in various centers on a large group of patients have shown the equivalence of the SentiMag^®^ method with the standard method with the use of a gamma camera and a radiotracer [9,10,11]. The SNB procedure is associated with the risk of postoperative complications, the type and frequency of which may differ depending on the SN detection method used [11,12,13].

The aim of the study was a retrospective, comparative assessment of complications of the SNB procedure in breast cancer using the radiotracer method as a reference and a relatively new SentiMag^®^ method on groups of patients after 3.5 years of use. The numbers of patients in both groups were comparable.

## 2. Materials and Methods

The study material was a group of 345 patients (median 62.3) with breast cancer, initially treated surgically, who underwent the SNB procedure with the use of a radiotracer. Procedures performed included: wide local excision (WLE), simple amputation (SA) and a stand-alone SNB procedure prior to induction treatment in the period from May 2018 to January 2021 at the Department of Oncological Surgery of the “K. Gibinski” University Clinical Center of the Medical University of Silesia in Katowice. The inclusion criteria for the study included 300 patients who were followed-up at the Hospital Outpatient Cancer Surgery Clinic and whose complications (paresthesia, limb mobility restriction and lymphoedema) were assessed. The longest follow-up period was 37 months, the shortest 5 months (mean 21 months). Before qualifying for the SNB procedure, the patients included in the study were examined and their medical history was taken at the Oncological Surgery Clinic, where the doctor assessed the limb mobility, measured the circumference and collected information about possible sensory disturbances. All patients also had ultrasound of the lymph nodes, and some of them had a fine needle biopsy (FNAB). Patients with unsuspected lymph nodes (c) N0 and no contraindications to the procedure were qualified for SN. The Technetium radiotracer was administered at the Department of Nuclear Medicine of our Center to the tumor area on the day preceding the procedure at approx. 10:00 a.m. Then, after performing lymphoscintigraphy, the patient was admitted to the Surgery Department. The surgical procedure was performed on the following day. The mean time from the administration of the radiotracer to the removal of SN was 18–20 h. Intraoperatively, SN was identified only by gamma camera, and no stain was used. The node with the highest pulse indication was considered SN. In addition, one or two additional nodes were collected, the signal of which were greater than 10% of the former. Each time in the operating protocol, the number of pulses in the identified SN node/nodes was recorded. After the procedure, for the first 2 years, the patients were checked at the Hospital Clinic every 3 months. In addition to the physical examination, ultrasound of the lymph nodes was performed. From the second to the fifth year after the procedure, a check-up was performed every 6 months. The control examination protocol also included the assessment of: (1) sensory disturbances in the form of paresthesia, (2) limb mobility limitation and (3) the presence of lymphedema.

A reduction in the range of motion by more than 20 degrees compared to the other limb was considered to be a significant limitation of limb mobility. Lymphedema was determined by measuring the circumference of the limb. A difference between the limbs of 10% was considered lymphedema: minimal (<20% difference), moderate (difference between 20% and 40%) and severe (>40% difference) [12].

The examination at the clinic was performed by the same team of four surgeons who operated on the patients. All of them have many years of experience in breast surgery. Patients who required armpit lymphadenectomy and/or interrupted the control cycle at the clinic were excluded from the study (31 patients required armpit lymphadenectomy, 14 interrupted the control cycle at the clinic).

**Figure 1:**
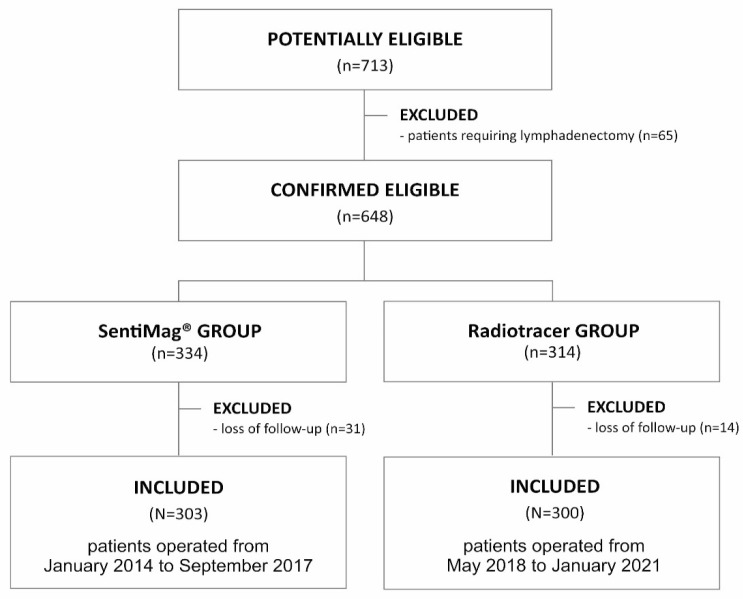
Flow diagram demonstrating the recruitment of the study group.

The analyzed group was compared in terms of the occurrence of the same complications with a similar (in terms of number of procedures—303) group of patients operated on in our center earlier in the period from January 2014 to September 2017, for whom SN identification was performed using the SentiMag^®^ method. The exclusion criteria were identical to the current study. The operations and control examinations were performed by the same team of surgeons. The recruitment process is shown in Figure 1. Results of this analysis were published in 2019 [13].

The Statistica 13.1 software (TIBCO Software Inc., Palo Alto, Santa Clara, CA, USA) and a MS Excel (Microsoft Corporation, Redmond, WA, USA) spreadsheet were used for the analysis. The non-parametric Mann–Whitney U test was used to establish the statistical significance of the differences between the incidence of complications and the number of removed lymph nodes. The significance of the remaining differences indicated in the multi-way tables was verified using the *χ*^2^ independence test. The level of statistical significance was adopted at the α level equal to 5%. Hence, *p* < 0.05 was adopted as the limit value of the probability of the test characteristics for which the differences were considered statistically significant.

## 3. Results

A total of 300 patients with SNB procedure were included in the study, with an age range of 29–87 years (mean 63 years). A total of 191 SNB procedures with wide local excision (WLE), 95 SNB procedures combined with simple amputation of the mammary gland (SA) and 14 standalone SNB procedures were performed prior to systemic treatment. In total, 864 lymph nodes were removed in all procedures. The SN identification rate in all procedures was 100%.

The mean of SN removed in one procedure with a radiotracer in the whole group was 2.9, (SentiMag^®^-2.9), with the standalone SNB procedure 3.2 (SentiMag^®^-1.8), with wide local excision (WLE) with SNB-2.4 (SentiMag^®^-2.5) and in simple amputation with SNB-3.2 (SentiMag^®^-3.6). The median follow-up was 26 months for WLE with SNB ((SentiMag^®^-25 months), 27 months for simple amputation with SNB (SentiMag^®^-26 months) and 11 months for SNB (SentiMag^®^-9 months) (Table 1).

Sensory disorders in the arm in the form of paresthesia occurred in 16 (14.1%) patients using the radiotracer method, SentiMag^®^-12 (11.2%), in 5 (2.6%) patients after WLE with SNB, SentiMag^®^-3 (1.5%), and 11 (11.5%) after SA with SNB, SentiMag^®^-9 (8.4%). 

Significant limitation of restricted upper limb mobility (ROM) was found in 12 (9.9%) SentiMag^®^ 9 (7.1%) patients, in 5 (2.6%) patients after WLE with SNB, SentiMag^®^-3 (1.5%), and in 7 (7.3%) after SA with SNB, SentiMag^®^-6 (5.6%).

Minimal grade lymphoedema occurred in 11 (11.58%) SentiMag^®^-9 (8.41%) patients, in 4 (2.09%) after WLE with SNB, SentiMag^®^-2 (1.05%), and 9 (9.47%) after SA with SNB, SentiMag^®^-7 (6.54%). 

In 14 (3.6%) patients, no complications were noted after the standalone SNB procedure, and, similarly, no complications were noted in the SentiMag^®^-5 (1.6%) (Table 2). 

The occurrence of paresthesia, restricted mobility and lymphedema was analyzed depending on the number of lymph nodes removed. A significant dependence of these complications on the number of lymph nodes removed in the SNB procedure in both methods was demonstrated (Table 3).

The analysis also included the occurrence of these complications depending on the type of procedure performed: SA and WLE. There was a significantly higher frequency of paresthesia and lymphoedema in SA with SNB compared to WLE with SNB in both methods. In the case of limb mobility restriction, no significant differences were observed depending on the type of surgery in both methods (Table 4).

The comparative analysis of the analyzed complications in the identification of SN did not show any significant differences in both methods (Table 5).

In the SentiMag^®^ method, skin discoloration was observed after the application of the Sienna +^®^ marker in the form of a brown-gray tattoo. The SentiMag^®^ method failed to detect the SN in two patients (0.5%) [13]. In the radiotracer method, SN was identified in all patients. In 34 patients (9.2%) in the SentiMag^®^ method and in 31 patients (8.9%) in the radiotracer method, histopathological examination of the material resulting from the SNB procedure revealed the presence of macrometastases in sentinel nodes, with infiltration of the capsule or the presence of neoplastic cells in the perinodular adipose tissue. These cases required an axillary lymphadenectomy and were excluded from the analysis.

## 4. Discussion

The sentinel node biopsy (SNB) procedure is the primary diagnostic method in the treatment of breast cancer for the evaluation of regional lymph node involvement on the path of lymphatic drainage from the tumor area. The result of this test makes it possible to decide on the treatment method. The method using a Technetium radiotracer (99 mTc) alone or in the double dye method is still considered the “gold standard” of node identification during the SNB procedure. The comparable effectiveness in this procedure has also been demonstrated using the SentiMag^®^ method, introduced a few years ago [9,14]. Complications may occur in both the Sienna +^®^ and radionuclide methods, but their frequency is much lower than in the case of axillary lymph node dissection (ALND).

A significant postoperative complication associated with SNB is lymphoedema, which occurs in 5–6% of patients subjected to this procedure. It may result in a reduction in the range of mobility of the upper limb, stiffness, numbness, as well as pain, reduction of self-esteem, secondary neoplasms and a tendency to withdraw from social life [12,15]

In our study, minimal grade lymphedema occurred in 13 out of 300 patients (4.3%) with the use of the radiotracer, and in 9 out of 303 patients (2.9%) with the SentiMag^®^ method (2.9%). Based on the collected data, there was no statistically significant difference in the incidence of lymphedema between SentiMag^®^ and the radiotracer method (*p*-0.3721). In our study, the application of the SNB procedure with the use of a radiotracer was associated with a low incidence of lymphedema: WLE with SNB (9.47%), SA with SNB (2.09%) and with standalone SNB (0%). Similarly, in the method using SentiMag^®^ for WLE with SNB (1.05%), for SA with SNB (6.54%) and standalone SNB (0%). In both methods, we demonstrated a statistically significant more frequent occurrence of lymphoedema with an increasing number of lymph nodes removed by simple amputation (SA) combined with SNB (*p* = 0.0000 and *p* = 0.0426). In cases of wide local excision (WLE) in combination with SNB, no significant differences were observed in both methods (*p* = 0.0785 and *p* = 0.1712). These differences probably result from the extent of the procedure. In simple amputation, an additional number of mute lymph nodes can be diagnostically removed along with Spence’s tail, which is clearly visible in the average number of removed nodes in both procedures (WLE with SNB—2.4 v. SA with SNB—3.2).

According to Aase Sagen et al. [16], the incidence of lymphedema 2.5 years after the surgery was 17% for the ALND procedure compared to 3% for the SNB procedure. In a large randomized clinical trial of NSABP B-32 [17], women with invasive breast cancer under-went SNB followed by ALND (group 1) or SNB followed by ALND only in the presence of sentinel nodal metastases (group 2). The 8-year overall and disease-free survival in both groups was found to be similar; the overall survival was 91.8% in Group 1 and 90.3% in Group 2, while the disease-free survival was 82.4% in Group 1 and 81.5% in Group 2. The authors concluded that with comparable survival in both of these treatments, it seemed reasonable to strive to remove as few lymph nodes as possible, and therefore routinely use the SNB procedure and possibly subsequent dissection of affected nodes, because this approach reduces the risk of lymphoedema in patients and, consequently, increases quality of life without affecting survival.

Another complication observed in our study was the presence of limitations in the mobility of the upper limb on the side of lymph node removal. They occurred after the use of the radiotracer in 12 out of 300 patients (4%) and in the SentiMag^®^ method in 9 out of 303 patients (2.9%). Again, a statistically significant correlation was demonstrated be-tween the frequency of mobility limitations and the number of surgically removed lymph nodes in both methods: radiotracer (WLE + SNB *p*-0002 and SA + SNB *p*-0.0000) and Sen-tiMag^®^ (WLE + SNB *p*-0.0307 and SA + SNB *p*-0.0426).

Paresthesia in the upper limb on the side of the procedure occurred with the use of a radiotracer in 16 out of 300 patients (5.3%) and with the use of superparamagnetics in 12 out of 303 patients (3.9%). In our study, paresthesia was significantly more frequent in the case of the SA + SNB procedure compared to the WLE + SNB procedure in both methods (radiotracer: *p*-0.0043; SentiMag^®^: *p*-0.0040). Despite the fact that in our study, no complications occurred for both methods after standalone SNB, other authors emphasized that despite the low invasiveness of this procedure, it may be followed by paresthesia, lymphoedema and limb mobility impairment [18]. As a result of applying the SentiMag^®^ method, a brown-gray skin discoloration was observed at the place where the Sienna+^®^ marker was administered. The discoloration receded completely after 1.5 year [13].

In the study by Ghilli M. et al. [19], the presence of tattoos was found in 47.3% of the studied group. Tattoo reduction occurred in 70.4% of patients and complete disappearance in 20.1% after an average of 5.9 months of follow-up in a group of 150 patients. In one (1.4%) patient the discoloration was enlarged, and in five (7.1%) patients the discoloration was not visibly reduced. On the other hand, in The Nordic SentiMag Trial [20], depigmentation occurred in 35.5% of patients and remained slightly paler and smaller after one year in 21% of patients and in 8.6% of patients after 15 months. In our ward, the SPIO injection was performed deep under the areola of the nipple, to a depth of 1–2 cm, resulting in a lower incidence of discoloration, which is also confirmed by reports by other authors [19,20]. The possibility of temporary discoloration should be the subject of information for patients undergoing this procedure.

In both methods, we demonstrated a similar effectiveness in terms of SN identification as well as a comparable risk of complications. Taking this into account and considering the fewer logistical difficulties associated with the use of the SentiMag^®^ method, the popularization of this method seems to be a direction worth considering in the diagnosis of regional lymph nodes in breast cancer. According to Ean-Louis Houpeau et al. [9], the use of superferromagnetism may turn out to be a good direction of development due to the possibility of the availability of nuclear reactors in Europe being limited, in which the radionuclide is produced. Another advantage of SPIO is the lack of a requirement for a nuclear medicine facility on the premises of a medical institution and absence of the transport difficulties typically associated with radioactive elements. Additionally, its use reduces the risk associated with exposure to ionizing radiation, both for staff and patients. Another relevant factor for both methods is their cost. The SentiMag is slightly more expensive than the standard radiomarker method.

The study is limited by the size of the sample as well as the fact that both the patient’s body mass index (BMI) and comorbidities were not taken into consideration.

## 5. Conclusions

SN identification using the radiotracer method and the SentiMag^®^ method are comparable diagnostic methods in breast cancer, with a low risk of complications.

## Figures and Tables

**Table 1:** General data.

The Total in the Particular Surgeries	Radiotracer			SentiMag^®^		
	WLE + SNB	SA + SNB	SNB	WLE + SNB	SA + SNB	SNB
Number of procedures	191	95	14	191	107	5
Age, years Average/median (range)	63.07/65 (36–89)	66.28/67 (33–89)	60.43/59 (45–75)	59.76/61 (30–84)	61.92/63 (40–88)	59.00/59 (47–71)
Removed lymph nodes Average/median (range)	2.47/2 (0–15)	3.22/2 (1–15)	3.29/2.5 (1–8)	2.53/2 (1–11)	3.61/3 (0–11)	1.83/1.5 (1–3)
Median of observations, months	26	27	11	25	26	9

**Table 2:** Complications in both methods.

Complication	Radiotracer			SentiMag^®^		
	WLE + SNB	SA + SNB	SNB	WLE + SNB	SA + SNB	SNB
Paresthesia, n (%)	5 (2.62)	11 (11.58)	0 (0.00)	3 (1.57)	9 (8.41)	0 (0.00)
Restricted upper limb mobility, ROM n (%)	5 (2.62)	7 (7.37)	0 (0.00)	3 (1.57)	6 (5.61)	0 (0.00)
Edema, n (%)	4 (2.09)	9 (9.47)	0 (0.00)	2 (1.05)	7 (6.54)	0 (0.00)

**Table 3:** Statistical significance (P) of differences between number of removed nodes depending on the occurrence of a complication and the kind on procedure in both methods (radiotracer and SentiMag).

Complication	Radiotracer			SentiMag^®^		
	WLE + SNB	SA + SNB	SNB	WLE + SNB	SA + SNB	SNB
Paresthesia	0.0272	0.0151	-	0.0293	0.0243	-
Restricted upper limb mobility, ROM	0.0002	0.0000	-	0.0307	0.0426	-
Edema	0.0785	0.0000	-	0.1712	0.0136	-

**Table 4:** The results of the chi independence test between the difference in the frequency of treatments (SA vs. WLE) depending on the complication occurrence for both methods (Radiotracer and Sentimag).

Complication	Radiotracer	SentiMag^®^
Paresthesia	χ^2^ (1; N = 286) = 9646; *p* = 0.002	χ^2^ (1; N = 298) = 8304; *p* = 0.004
Restricted upper limb mobility, ROM	χ^2^ (1; N = 286) = 3562; *p* = 0.059	χ^2^ (1; N = 298) = 3816; *p* = 0.051
Edema	χ^2^ (1; N = 286) = 7963; *p* = 0.005	χ^2^ (1; N = 298) = 707; *p* = 0.008

**Table 5:** Statistical significance of differences in the number of complications between the methods with the radiotracer (n = 300) and SentiMag^®^ (n = 303).

Complication	*p* Value	*χ*^2^ Test Result
Paresthesia	0.4231	0.642
Restricted upper limb mobility, ROM	0.4905	0.476
Edema	0.3721	0.797

## Data Availability

Data is available upon special request.
